# Identification and Expression Analysis of *CAMTA* Genes in Tea Plant Reveal Their Complex Regulatory Role in Stress Responses

**DOI:** 10.3389/fpls.2022.910768

**Published:** 2022-05-27

**Authors:** Qiying Zhou, Mingwei Zhao, Feng Xing, Guangzhi Mao, Yijia Wang, Yafeng Dai, Minghui Niu, Hongyu Yuan

**Affiliations:** ^1^Henan Key Laboratory of Tea Plant Biology, Xinyang Normal University, Xinyang, China; ^2^Henan Engineering Research Center of Tea Deep-Processing, Xinyang Normal University, Xinyang, China; ^3^Institute for Conservation and Utilization of Agro-Bioresources in Dabie Mountains, Xinyang Normal University, Xinyang, China

**Keywords:** tea plant, genome-wide, expression analysis, *CAMTA*, gene family

## Abstract

Calmodulin-binding transcription activators (CAMTAs) are evolutionarily conserved transcription factors and have multi-functions in plant development and stress response. However, identification and functional analysis of tea plant (*Camellia sinensis*) *CAMTA* genes (*CsCAMTAs*) are still lacking. Here, five *CsCAMTAs* were identified from tea plant genomic database. Their gene structures were similar except *CsCAMTA2*, and protein domains were conserved. Phylogenetic relationship classified the CsCAMTAs into three groups, CsCAMTA2 was in group I, and CsCAMTA1, 3 and CsCAMTA4, 5 were, respectively, in groups II and III. Analysis showed that stress and phytohormone response-related *cis*-elements were distributed in the promoters of *CsCAMTA* genes. Expression analysis showed that *CsCAMTAs* were differentially expressed in different organs and under various stress treatments of tea plants. Three-hundred and four hundred-one positive co-expressed genes of *CsCAMTAs* were identified under cold and drought, respectively. *CsCAMTAs* and their co-expressed genes constituted five independent co-expression networks. KEGG enrichment analysis of *CsCAMTAs* and the co-expressed genes revealed that hormone regulation, transcriptional regulation, and protein processing-related pathways were enriched under cold treatment, while pathways like hormone metabolism, lipid metabolism, and carbon metabolism were enriched under drought treatment. Protein interaction network analysis suggested that CsCAMTAs could bind (G/A/C)CGCG(C/G/T) or (A/C)CGTGT *cis* element in the target gene promoters, and transcriptional regulation might be the main way of *CsCAMTA*-mediated functional regulation. The study establishes a foundation for further function studies of *CsCAMTA* genes in stress response.

## Introduction

Calcium (Ca^2+^) is a well-known second messenger that can be activated by various exogenous and endogenous signals (Zhang et al., 2014; Furio et al., 2020). Then, the signals are relayed by downstream proteins, including calmodulins/calmodulin-like proteins (CaMs/CMLs), calcium-dependent protein kinases (CDPKs), and calcineurin B-like proteins (CBLs), to regulate gene expression and different biochemical processes (Rahman et al., 2016; Wei et al., 2017). Calmodulin-binding transcription activators (CAMTAs) are a kind of novel and well-studied CaM-interacting transcription factors and are evolutionarily conserved in almost all eukaryotes (Yang et al., 2015; Kakar et al., 2018). After being identified in tobacco, genome-wide identification and analysis of *CAMTA* genes have been done in many plant species, including Arabidopsis (*Arabidopsis thaliana*; Bouché et al., 2002), rice (*Oryza sativa*; Choi et al., 2005), maize (*Zea mays*; Yue et al., 2015), soybean (*Glycine max*; Wang et al., 2015), grape (*Vitis vinifera*; Shangguan et al., 2014), cotton (*Gossypium* spp.; Pant et al., 2018), medicago (*Medicago truncatula*; Yang et al., 2015), citrus (*Citrus sinensis* and *Citrus clementina*; Zhang et al., 2019a), and poplar (*Populus trichocarpa*; Wei et al., 2017).

CAMTA proteins are characterized by containing several conserved functional domains from the N terminus to the C terminus, including CG-1 domain, TIG/IPT (transcription-associated immunoglobulin domain/immunoglobulin-like fold shared by plexins and transcription factors) domain, ankyrin repeat (ANK) domain, IQ domain (IQXXXRGXXXR), and calmodulin-binding domain (CaMBD). The CG-1 domain is a specific DNA binding domain, usually with nuclear localization signal (NLS) at N terminus. The TIG/IPT domain is responsible for non-specific DNA-binding. The ANK domain is evolved to mediate protein–protein interactions. The IQ domain is Ca^2+^-independent CaM interacting domain, while the CaMBD domain is Ca^2+^-dependent CaM binding domain (Yang et al., 2012; Rahman et al., 2016; Büyük et al., 2019). By binding with the (G/A/C)CGCG(C/G/T) or (A/C)CGTGT *cis* element in the promoter region, CAMTA proteins thus can interact and regulate the transcription of target genes (Yue et al., 2015; Noman et al., 2019; Sun et al., 2020).

CAMTA protein is a pivotal component of the Ca^2+^ signal transduction pathway and plays important roles in plant growth and development, as well as in an array of stress responses (Yang et al., 2012; Shangguan et al., 2014; Zhang et al., 2019a). Tobacco (*Nicotiana tabacum*) *NtER1*, the first *CAMTA* gene identified in plant, was a trigger for senescence and death, with high expression in senescing tissues (Yang et al., 2015). AtCAMTA1 and AtCAMTA5 were important regulators in *Arabidopsis* pollen development by affecting the expression of *AVP1* gene, which functioned in the vacuolar pH maintenance (Zhang et al., 2019a). Recent research showed that AtCAMTA6/SR3 was important for seed germination under salt stress environment (Shkolnik et al., 2019). *CAMTA* genes also exhibited dynamic expression patterns in different organs and development stages and was related to fruit ripen (Yang et al., 2012; Ouyang et al., 2019). *AtCAMTA1*, *AtCAMTA2,* and *AtCAMTA3* could regulate plant cold tolerance together by inducing the expression of *CBF* genes (Doherty et al., 2009; Kim et al., 2013; Novikova et al., 2020). *CAMTA* gene was also contributed to plant drought resistance (Pandey et al., 2013; Meer et al., 2019; Saeidi et al., 2019). Expression of *citrus CAMTA* gene was upregulated under salt stress, indicating a role of *CAMTA* gene in salt stress (Ouyang et al., 2019). AtCAMTA3 was proved to regulate glucose metabolism and ethylene-induced senescence and also important for plant defense against insect herbivores (Qiu et al., 2012; Yang et al., 2015). Recently, *AtCAMTA1*, *AtCAMTA2,* and *AtCAMTA3* were reported to participate in plant immune reactions (Kim et al., 2020; Sun et al., 2020). In addition, *CAMTA* gene could respond to plant hormone signals and display a close relationship with ethylene (ET), SA (salicylic acid) and JA (jasmonic acid; Yang et al., 2015; Yue et al., 2015; Wei et al., 2017).

Tea is one of the three most popular non-alcoholic beverages in the world and is made from leaves of tea plant (*Camellia sinensis*). In recent years, gene function studies and role of transcription factors have been focused in tea plant science (Wei et al., 2018; Zhang et al., 2020). As a kind of sessile organism, tea plant is vulnerable to external environmental stresses, such as cold, drought, salt, and pest attack (Zhang et al., 2017; Li et al., 2019; Zhou et al., 2019a). CAMTAs, one kind of important regulator in plant growth, development, and stress response, have not been identified and studied in tea plant to date. In this study, tea plant *CAMTA* (*CsCAMTA*) genes were identified from the reported genomic database (Wei et al., 2018). The phylogenetic relationship, gene structure, promoter *cis* elements of *CsCAMTA* genes, and conserved CsCAMTA protein domains were analyzed. *CsCAMTA* gene expression profiles in different organs and under different stress treatments, co-expression networks of *CsCAMTA* genes under cold and drought stresses, and KEGG enrichment analysis of *CsCAMTA*s and their co-expressed genes were done. Also, interaction network of CsCAMTA proteins in tea plant was built, and the putative binding sites in the target genes were identified. This work provides the first survey of *CsCAMTA* genes in tea plant and is important for further function studies of *CsCAMTA* genes and tea plant resistance improvement.

## Materials and Methods

### Identification and Analysis of Putative *CsCAMTA* Genes in Tea Plant

The whole genomic sequence data of tea plant were downloaded from the State Key Laboratory of Tea Plant Biology and Utilization^1^ according to Wei et al. (2018). The hidden Markov models (HMMs) of CAMTA family (Pfam03859, CG-1 domain; Pfam01833, TIG domain; Pfam12796, Ank domain; Pfam00612, IQ domain) were used to identify *CsCAMTA* genes from the tea plant genomic database by HMMER (V3.3) software. Then, a BLASTp program was used to search the tea plant protein database by employing the amino acid sequences of *Arabidopsis* CAMTAs as a query. At last, the putative CsCAMTAs identified by the HMMER and BLASTp programs were put together. Redundant CsCAMTAs were removed, and the remaining putative CsCAMTAs were further confirmed by domain analysis using the CDD (Conserved Domain Database^2^) and SMART^3^ programs. Those containing CG-1 domain, TIG domain, Ank domain, and IQ domain were recognized as *CsCAMTA* gene family members.

Molecular weights (MW) and isoelectric points (pI) of the recognized CsCAMTA proteins were computed by the Compute pI/Mw tool of ExPASy.^4^ The physicochemical properties of CsCAMTA proteins were computed by ProtParam Tool.^5^ The most similar homolog of each *CsCAMTA* gene was analyzed by search against the Araport11 transcripts (DNA) database using BLASTN program, and the expect value (E-value) was given after the alignment. Subcellular locations of the identified CsCAMTA proteins were predicted using the CELLO v.2.5^6^ online tool.

### Phylogenetic Relationship Analysis

CAMTA protein sequences of *Arabidopsis*, *Oryza sativa* (ssp. Japonica), *Vitis vinifera*, *Populus trichocarpa*, and *Citrus clementina* were downloaded from the PlantTFDB v5.0 database.^7^ CAMTA proteins in *Arabidopsis* and *Populus trichocarpa* were named according to Doherty et al. (2009) and Wei et al. (2017), respectively. Multiple sequence alignment was done by using the Clustalw program. The phylogenetic tree construction was accomplished by the MEGA 6.0 software with the neighbor joining method, and the bootstrap was set as 1,000 replications (Tamura et al., 2013).

### Gene Structure and Sequence Analysis

The transcript sequences, DNA sequences, and protein sequences of *CsCAMTA* genes were obtained from the tea plant genomic database. The conserved domains of CsCAMTA proteins were identified in Pfam database. NLS of the CsCAMTA proteins was analyzed by Motif scan.^8^ The calmodulin binding domain (CaMBD) sequences of CsCAMTA proteins were identified manually according to the conserved motif sequence (WXVX(2)LXKX(2)[LF]RWRX[KR]X(3)[FL]RX) of CAMTA protein in *Arabidopsis* (Pant et al., 2018). Conserved domains, gene structures, and the CaMBD sequence logo of the CsCAMTAs were visualized by the TBtools toolkit (Chen et al., 2020).

### Promoter *cis*-Elements Analysis of *CsCAMTA* Genes

To investigate the promoter *cis*-elements of *CsCAMTA* genes, 2.0 kb genomic DNA sequences upstream of the initiation codon (ATG) were retrieved from the Tea Plant Information Archive (TPIA; Xia et al., 2019). Stress response and hormone regulation related *cis*-elements were predicted by PlantCARE.^9^

### Analysis of *CsCAMTA* Gene Expression by RNA-seq Data

The RNA-seq data of tea plant different tissues (Wei et al., 2018) and those of tea plants under cold acclimation treatments (Li et al., 2019), under NaCl treatment and drought stress treatment (Zhang et al., 2017) and under MeJA treatment (Shi et al., 2015), were, respectively, downloaded from the public database according to the cited literature. Gene expression level was represented by the FPKM (Fragments per kilobase of transcript sequence per millions fragments mapped) value using the Tophat and Cufflink softwares (Trapnell et al., 2010). The expression heatmaps were generated and visualized by the TBtools toolkit (Chen et al., 2020).

### Gene Co-expression Network and KEGG Analysis

To understand the co-expression network of *CsCAMTA* genes under cold and drought stresses, positive co-expressed genes of *CsCAMTAs* were retrieved from the Tea Plant Information Archive according to the Pearson’s correlation based on the TPM (Transcripts per million) value of the transcriptomic data (TPIA; Xia et al., 2019). For the co-expression network of *CsCAMTA* genes under cold stress, the threshold of Pearson’s correlation was ≥0.98, with *value of p* ≤ 0.05. Under PEG-simulated drought stress, the threshold of Pearson’s correlation was ≥0.98, *value of p* was ≤0.001. Then, Cytoscape (version 3.7.2) software was employed to visualize the co-expression networks.

Protein sequences of *CsCAMTAs* and their co-expressed genes were extracted from the tea genomic database (Wei et al., 2018). The KO IDs of CsCAMTAs and their co-expressed gene-coding proteins were acquired by mapping the protein sequences to the KEGG database *via* KAAS. The significantly enriched pathways were analyzed by the KOBAS software with value of *p* ≤ 0.05.

### Protein Interaction Network Analysis

For protein interaction network analysis, CsCAMTA protein orthologs in *Arabidopsis* were used and analyzed by employing the online STRING tools.^10^

Tea plant homologs of the *Arabidopsis* proteins displayed in the interaction network were identified by BLASTp program. Then, 2.0 kb genomic DNA sequences upstream of the initiation codon (ATG) of the identified tea plant homologous genes were retrieved from the Tea Plant Information Archive (TPIA; Xia et al., 2019). The conserved CAMTA binding *cis* elements (G/A/C)CGCG(C/G/T) and (A/C)CGTGT were examined in the 2.0 kb upstream genomic region of the identified tea plant homologous genes.

### Plant Materials and Treatments

Xinyang “Quntizhong” were cultivated in the Experimental Tea Garden of Xinyang Normal University. For tissue expression pattern analysis, apical buds, the first and the second leaves downward from the apical bud (young leaves), mature leaves, old leaves, stems, flowers, fruits, and roots were collected from the same Xinyang “Quntizhong.” Tender branches (about 15 cm long) from the same “Quntizhong” tea plant were collected and then used for treatments. For cold treatment, the branches were inserted into the tissue culture bottles with 0.3 cm depth of water and placed in 4°C culture chamber, 70% relative humidity, and 12-h photoperiod with 100 μmol m^−2^ s^−1^ light intensity, while the controls were placed at the tissue culture room at 25°C, 70% relative humidity, and 12-h photoperiod with 100 μmol m^−2^ s^−1^ light intensity. For NaCl treatment, the tea plant branches were inserted into the tissue culture bottles with 300 mM NaCl. For PEG-simulated drought treatment, the tea plant branches were inserted into the tissue culture bottles with 10% PEG6000. For MeJA treatment, the tea plant branches were inserted into the tissue culture bottles with 1 mM MeJA solution which was dissolved in ethanol. The final concentration of ethanol in 1 mM MeJA was 0.5%. For the NaCl and PEG-simulated drought treatments, tea plant branches inserted into the tissue culture bottles with deionized water were used as controls. For the MeJA treatment, tea plant branches inserted into the tissue culture bottles with 0.5% ethanol were used as controls. Both the treatments and controls were placed in the tissue culture room at 25°C, 70% relative humidity, and 12-h photoperiod with 200 μmol m^−2^ s^−1^ light intensity. One to three leaves downward from the apical bud were, respectively, collected after 0, 3, 6, 12, 24, and 48 h of each treatment. For different tea plant tissues and each treatment, materials of three independent biological repeats were collected. The collected plant materials were frozen in liquid nitrogen quickly and stored in −80°C for RNA extraction.

### qRT-PCR Analysis

Total RNAs of the collected tea plant materials were, respectively, isolated by the modified CTAB method (Zhou et al., 2019a). Then, cDNA of each sample was synthesized from the total RNA by using the PrimeScript^™^ RT Reagent Kit (Takara) following the manufacturer’s instructions. qRT-PCR analysis was carried out on the LightCycler 96 real-time PCR instrument (Roche) using BrightGreen 2 × qRT-PCR MasterMixes (ABM, Canada). qRT-PCR amplification was as follows: 95.0°C 3 min, followed by 40 cycles of 95.0°C 15 s, 20 s at the primer-specific annealing temperature, and 72.0°C 30 s. Melting curve was generated by heating from 65°C to 95°C in 0.5°C increments to check the primer specificity. The glyceraldehyde-3-phosphate dehydrogenase gene of tea plant (*CsGAPDH*) was used as the internal reference gene, and the relative gene expression was calculated according to the 2^−ΔΔCt^ method (Zhou et al., 2019a). Specific primers of *CsCAMTA* genes and the *CsGAPDH* gene were designed by primer 3.0 and listed in Supplementary Table S1.

## Results

### Genome-Wide Identification of *CAMTA* Gene in Tea Plant

By BLASTp and hidden Markov models (HMMs) search of the tea plant genomic database, the returned CsCAMTAs were checked again by the CDD (Conserved Domain Database) and the SMART programs for identifying the conserved domains of CAMTA protein. In total, five *CsCAMTA* genes containing the CG-1 domain, TIG domain, Ank domain, and IQ domain were identified, which were named *CsCAMTA1-5*, respectively. Details of the identified *CsCAMTA* genes, such as gene ID, amino acid number, molecular weight, isoelectric point (pI), instability index, grand average of hydropathicity (GRAVY), aliphatic index, the *Arabidopsis* homolog, E-value of each *CsCAMTA* gene compared with its corresponding *Arabidopsis* homolog, and subcellular localization of CsCAMTAs, are summarized in Table 1. The deduced CsCAMTA protein lengths were varied from 864 to 1,419 amino acids, with the molecular weights ranged from 97.26 to 157.99 kDa, and the predicted isoelectric points were ranged from 5.83 to 6.55. As shown in Table 1, the GRAVY values of all the identified CsCAMTA proteins were negative, indicating they were hydrophilic proteins. The instability index value above 40 was predicted as unstable. Results showed that the instability index value of CsCAMTA2 was below 40 while those of the other CsCAMTA proteins were above 40 (Table 1), indicating the instability of CsCAMTAs except CsCAMTA2. The predicted aliphatic indexes suggested high thermal stability of CsCAMTA proteins, with CsCAMTA2 having the highest aliphatic index of 82.76 and CsCAMTA3 having the lowest aliphatic index of 74.16. When the *AtCAMTA* homologs were identified, it showed that *AtCAMTA4* was the homolog of *CsCAMTA1* and *CsCAMTA3* genes, *AtCAMTA3* was the homolog of *CsCAMTA2* gene, and *AtCAMTA5* was the homolog of *CsCAMTA4* and *CsCAMTA5* genes, with the smallest E-value. Subcellular localization prediction showed that all CsCAMTA proteins were located in cell nucleus, and CsCAMTA2 and CsCAMTA5 also had a cytoplasmic localization (Table 1).

**Table 1:** The *CAMTA* genes in tea plant and properties of the deduced proteins.

Name	Gene ID	Amino acids size (aa)	MW (kDa)	pI	GRAVY	Instability index	Aliphatic index	*Arabidopsis* homolog	*E*-value	Subcellular location
*CsCAMTA1*	*TEA025813.1*	1,028	114.8	6.08	−0.516	46.14	79.14	*AtCAMTA4*	2.00E-151	Nuclear
*CsCAMTA2*	*TEA012477.1*	1,419	157.99	6.41	−0.345	39.39	82.76	*AtCAMTA3*	2.00E-88	Nuclear, Cytoplasmic
*CsCAMTA3*	*TEA006574.1*	984	109.83	5.93	−0.516	45.8	74.16	*AtCAMTA4*	0	Nuclear
*CsCAMTA4*	*TEA011186.1*	953	107.62	6.55	−0.468	44.04	75.14	*AtCAMTA5*	7.00E-106	Nuclear
*CsCAMTA5*	*TEA011490.1*	864	97.26	5.83	−0.39	43.38	77.27	*AtCAMTA5*	7.00E-105	Nuclear, Cytoplasmic

### *CsCAMTA* Gene Structure and Conserved Domains Analysis

**Figure 1:**
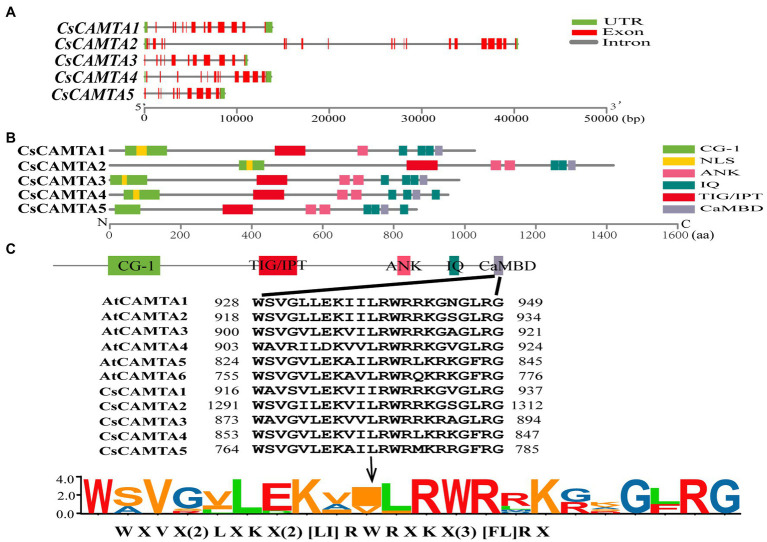
Gene structure, protein functional domains, and CaMBD domain conservation analysis of CsCAMTA family. **(A)** The exon–intron organization of *CsCAMTA* genes. Exons and introns are, respectively, represented by red boxes and gray lines. The untranslated region (UTR) is indicated by green boxes. **(B)** Schematic representation of functional domains of CsCAMTA proteins. CG-1, DNA binding domain; NLS, nuclear localization signal; ANK, ankyrin repeats; IQ, Ca^2+^-independent CaM-interacting domain; TIG/IPT, non-specific DNA binding domain; CaMBD, Ca^2+^-dependent CaM binding domain. **(C)** CaMBD sequence alignment and the CaMBD sequence logo of CsCAMTA and AtCAMTA proteins. “[]” suggests the amino acids allowed in this position of the CaMBD motif; “X” denotes any amino acid, and “()” indicates the number of amino acids.

The exon–intron structure of *CsCAMTA* genes was analyzed by mapping the transcript sequences to the corresponding genomic sequences. Results showed that most of the *CsCAMTA* genes had similar gene structures, with 10–12 introns. Except that *CsCAMTA2* gene contained 19 introns, and *CsCAMTA4* gene contained 13 introns, among which, one located in the 5′ untranslated region (Figure 1A). To better understand the potential functions of CsCAMTAs, conserved domains and NLS of the CsCAMTAs were analyzed. Results displayed that all CsCAMTA proteins contained one CG-1 and one TIG/IPT domain, while more than one ANK and IQ domains were contained in most of the CsCAMTA proteins. The NLS was identified in most of the CsCAMTA proteins except CsCAMTA5, and it was laid inside the CG-1 domain (Figure 1B). The CaMBD was identified in all of the five CsCAMTAs (Figure 1B), it had a consensus sequence of WXVX(2)LXKX(2)[LI]RWRXKX(3)[FL]RX (Figure 1C), which was very similar to that in *Arabidopsis* and tomato (Bouché et al., 2002; Pant et al., 2018).

### Phylogenetic Analysis of *CsCAMTA* Genes

**Figure 2:**
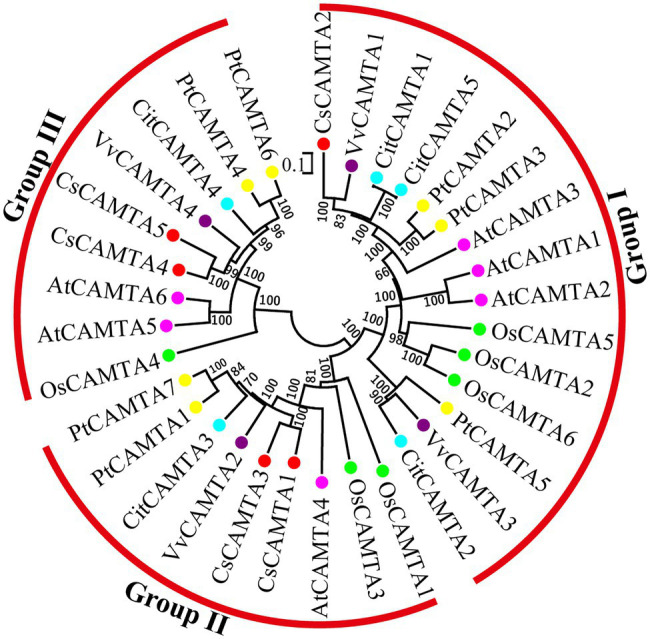
The phylogenetic tree of CAMTA proteins from *Camellia sinensis* (CsCAMTA), *Arabidopsis* (AtCAMTA), *Oryza sativa* (OsCAMTA), *Vitis vinifera* (VvCAMTA), *Populus trichocarpa* (PtCAMTA), and *Citrus clementine* (CitCAMTA) was built by MEGA 6.0 software. Bootstrap values are displayed beside the branches. Dots with different colors represented CAMTA proteins of different plant species (Red, *Camellia sinensis*; Purple, *Vitis vinifera*; Blue, *Citrus clementine*; Yellow, *Populus trichocarpa*; Pink, *Arabidopsis*; Green, *Oryza sativa*).

To investigate the phylogenetic relationship of *CsCAMTA* genes with those in other plants, a phylogenetic tree containing 33 CAMTA proteins from tea plant, arabidopsis, rice, grape, poplar, and citrus was built. Information of the CAMTA proteins from the six plant species was provided in Supplementary Table S2. Results showed that the CAMTA proteins could be clustered into three groups (I, II, and III); each group had the CsCAMTA protein member (Figure 2). Group I was the largest one, with 15 members from six plant species, including CsCAMTA2, AtCAMTA1, AtCAMTA2, and AtCAMTA3. There were 9 members in both group II and III, respectively. CsCAMTA1, CsCAMTA3, and AtCAMTA4, together with two CAMTAs in rice (OsCAMTA1 and OsCAMTA2), two CAMTAs in poplar (PtCAMTA1 and PtCAMTA7), VvCAMTA2 in grape, and CitCAMTA3 in citrus were belonged to group II. CsCAMTA4, CsCAMTA5, along with AtCAMTA5, AtCAMTA6, grape VvCAMTA4, citrus CitCAMTA4, poplar PtCAMTA4 and PtCAMTA6, and rice OsCAMTA4 were presented in group III (Figure 2).

### *Cis* Elements in the Promoters of *CsCAMTA* Genes

**Figure 3:**
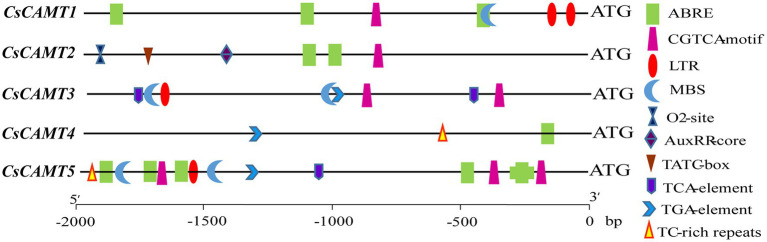
Stress and phytohormone-response related *cis* elements in the promoter of *CsCAMTA* genes.

To understand the putative function of *CsCAMTA* genes in tea plant, the promoter *cis*-elements of *CsCAMTA* genes were investigated. Results revealed that *cis*-elements related to stress and hormone response were found in the promoter of *CsCAMTA* genes (Figure 3; Supplementary Table S3). *CsCAMTA5* gene had the most number of stress and hormone response-related *cis*-elements in its promoter, whereas *CsCAMTA4* gene had the least. The LTR (low-temperature responsiveness) and the MBS (MYB binding site involved in drought-inducibility) *cis*-elements were existed in all *CsCAMTA* gene promoters except those of *CsCAMTA2* and *CsCAMTA4*. The TC-rich repeat (involved in defense and stress responsiveness) was identified in the promoters of *CsCAMTA4* and *CsCAMTA5*. The CGTCA-motif (involved in the MeJA-responsiveness) was found in all *CsCAMTA* gene promoters except that of *CsCAMTA4*. The TCA element (involved in salicylic acid responsiveness) was found in the promoters of *CsCAMTA3* and *CsCAMTA5*. The ABRE element was found in all of the identified *CsCAMTA* gene promoters except that of *CsCAMTA3*. The O_2_-site (involved in zein metabolism regulation), TATC element (involved in gibberellin-responsiveness), and AuxRR core element (involved in auxin responsiveness) were specifically found in the *CsCAMTA2* gene promoter (Figure 3; Supplementary Table S3).

### Expression Profiles of *CsCAMTA* Genes in Different Organs and Under Different Abiotic Stresses

**Figure 4:**
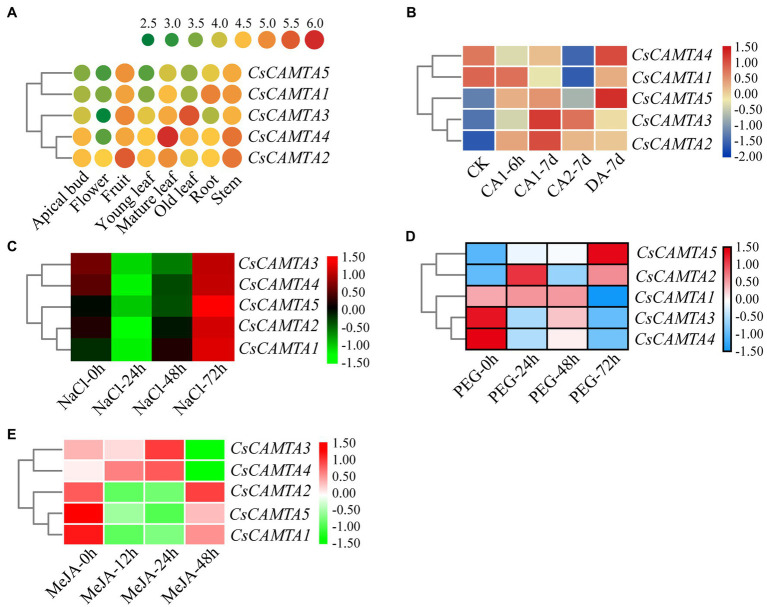
*CsCAMTA* gene expression pattern analysis by publicly available RNA-seq data. **(A–E)** Respectively, denote the expression pattern of *CsCAMTA* genes in different tea plant tissues, under cold acclimation, under salt stress, under PEG-simulated drought stress, and after MeJA treatments. CK, control conditions (Day/night, 25°C 12 h/20°C 12 h); CA1, cold acclimation (Day/night, 10°C 12 h/4°C 12 h); CA2, cold acclimation (Day/night, 4°C 12 h/0°C 12 h); DA, de-acclimation (Day/night, 25°C 12 h/20°C 12 h); NaCl, 200 mM sodium chloride; PEG, 25% polyethylene glycol; MeJA, 0.25% (v/v) methyl jasmonate.

**Figure 5:**
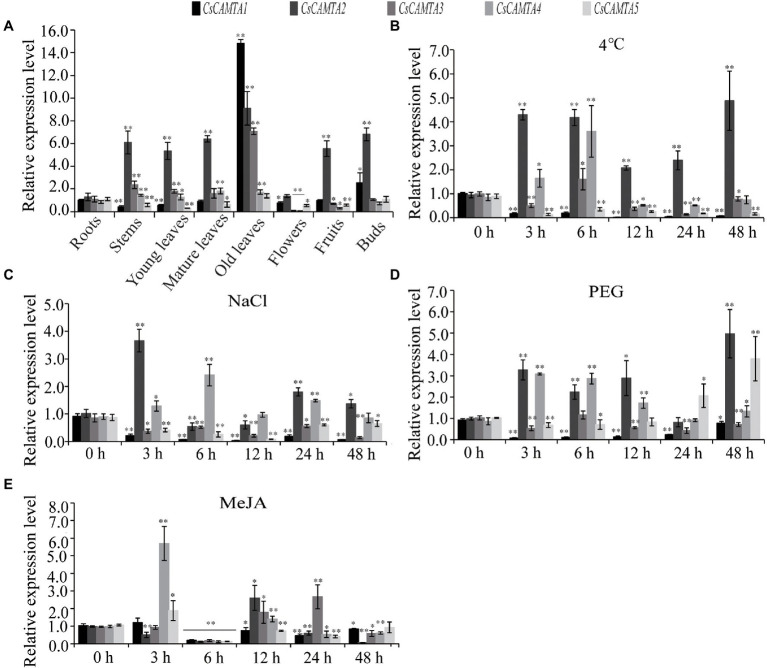
*CsCAMTA* gene expression pattern by qRT-PCR analysis. **(A–E)** Respectively, denote the expression pattern of *CsCAMTA* genes in different tea plant tissues, under cold treatment, under NaCl treatment, under PEG treatment, and under MeJA treatment. The relative expression level was normalized using that of *CsGAPDH* gene. Values are the mean and SD of three biological replicates. Statistical analysis was the Student’s *t*-test, ^*^p < 0.05 and ^**^p < 0.01.

Tissue-specific expression analysis would help us to understand the roles of *CsCAMTA* genes. By employing the publicly available RNA-seq data, tissue-specific expression patterns of *CsCAMTA* genes were displayed with heatmap according to the FPKM values (Figure 4A). Results showed that *CsCAMTA2* and *CsCAMTA4* genes relatively had higher expression level in almost all tissues, and *CsCAMTA2* had the highest expression level in fruit, *CsCAMTA4* had the highest expression level in mature leaf. *CsCAMTA3* displayed the highest expression level in old leaf, and its expression was relatively lower in flower and root. *CsCAMTA1* and *CsCAMTA5* genes displayed higher expression in root, stem, and fruit and lower expression level in flower and different developmental stages of leaf tissues (Apical bud, Yong leaf, Old leaf; Figure 4A). Further qRT-PCR analysis showed that *CsCAMTA2* and *CsCAMTA4* genes had relative higher expression level in almost all the vegetative tissues examined; the *CsCAMTA2* gene also showed higher expression level in fruits, when compared with the expression level in roots (Figure 5A). *CsCAMTA3* gene displayed higher expression in stems, young leaves, and old leaves and lower expression in flowers and fruits, compared with its expression in roots (Figure 5A). Except higher expression of *CsCAMTA1* gene was shown in buds and old leaves, the expression levels of *CsCAMTA1* and *CsCAMTA5* genes were lower in the measured tissues, when compared with that in the roots (Figure 5A).

To understand functions of *CsCAMTA* genes in stress response, expression profiles of *CsCAMTA* genes were analyzed using the public RNA-seq data first. After cold acclimation (CA) treatment, results showed that expression levels of *CsCAMTA2*, *CsCAMTA3*, and *CsCAMTA5* were upregulated, while those of *CsCAMTA1* and *CsCAMTA4* were downregulated, when compared with that of control (CK). *CsCAMTA4* and *CsCAMTA5* displayed the highest expression level in tea plant after the de-acclimation treatment (DA; Figure 4B). qRT-PCR analysis showed that the expression of *CsCAMTA2* gene was upregulated after cold treatment. *CsCAMTA3* and *CsCAMTA4* genes also displayed upregulation after 6 h of the cold treatment, but their expression was downregulated after 12 and 24 h of the cold treatment (Figure 5B). The expression levels of *CsCAMTA1* and *CsCAMTA5* genes were significantly downregulated after cold treatment (Figure 5B). Under NaCl treatment, RNA-seq data analysis showed that expression levels of all the *CsCAMTA* genes were downregulated after 24 and 48 h of the treatment, except that the expression of *CsCAMTA1* gene was increased after 48 h of the treatment, when compared with the expression level at 0 h. However, expression levels of all the *CsCAMTA* genes were upregulated after 72 h of the NaCl treatment (Figure 4C). By qRT-PCR analysis, results showed that the expression of *CsCAMTA1*, *3,* and *5* genes was downregulated after both the 24 and 48 h of NaCl treatment (Figure 5C). The expression of *CsCAMTA4* gene was upregulated after 24 h and had no obvious change after 48 h of NaCl treatment, while the expression of *CsCAMTA4* gene was upregulated after 24 and 48 h of NaCl treatment (Figure 5C). After PEG-simulated drought treatment, RNA-seq data analysis showed that the expression levels of *CsCAMTA3* and *CsCAMTA4* were downregulated, and that of *CsCAMTA1* showed no obvious change after 24 and 48 h, but was downregulated after 72 h of the PEG treatment (Figure 4D), while those of *CsCAMTA2* and *CsCAMTA5* were upregulated. Expression of *CsCAMTA2* and *CsCAMTA5* was upregulated after 24 h of the PEG treatment. The expression of *CsCAMTA5* gene exhibited a continuous increase, and it displayed the highest expression level at 72 h of the treatment (Figure 4D). qRT-PCR analysis showed that the expression of *CsCAMTA1* and *CsCAMTA3* genes was downregulated, while that of *CsCAMTA2* and *CsCAMTA4* genes was upregulated, after PEG treatment (Figure 5D). The expression of *CsCAMTA5* gene was downregulated after 3 and 6 h and was upregulated after 24 and 48 h after PEG treatment (Figure 5D). Expression of *CsCAMTA* genes under MeJA treatment was also investigated, RNA-seq data analysis showed that expression levels of *CsCAMTA1*, *CsCAMTA2,* and *CsCAMTA5* were downregulated, and that of *CsCAMTA4* was upregulated after 12 and 24 h of the treatment. Expression level of *CsCAMTA3* gene was downregulated after 12 and upregulated after 24 h of the MeJA treatment. After 48 h of the treatment, expression of all the *CsCAMTA* genes was decreased, except that of *CsCAMTA2* showed a little increment, when compared with the expression level at 0 h (Figure 4E). After MeJA treatment, qRT-PCR analysis showed that the expression of *CsCAMTA1* and *5* was downregulated after 12 and 24 h, and that of *CsCAMTA2* and *4* was upregulated after 12 h and downregulated after 24 h (Figure 5E). The expression of *CsCAMTA3* was upregulated after both 12 and 24 h of MeJA treatment (Figure 5E). After 48 h of MeJA treatment, except the expression of *CsCAMTA5* gene showed no obvious change, that of the other *CsCAMTA* genes was downregulated (Figure 5E).

### Functional Annotation and Co-expression Network of *CsCAMTA* Genes Under Cold and Drought Treatments

To understand the putative functions of *CsCAMTA* genes in tea plant, functional annotation of *CsCAMTA* genes was investigated in GO, KEGG, TrEMBL, Swissprot, and Nr database, respectively. Results showed that the five identified *CsCAMTA* genes were annotated as calmodulin-binding transcription activators in TrEMBL, Swissprot, and Nr databases. According to GO and KEGG annotation, *CsCAMTA1* and *CsCAMTA3* were annotated as proteins related to lipase, ester hydrolase, LPA acyltransferase, and signal recognition; *CsCAMTA4* and *CsCAMTA5* were related to transcription regulation, motor protein, and mitosis, and *CsCAMTA4* was also involved in lipid metabolism; *CsCAMTA2* was annotated as MLO family protein (Supplementary Table S4).

**Figure 6:**
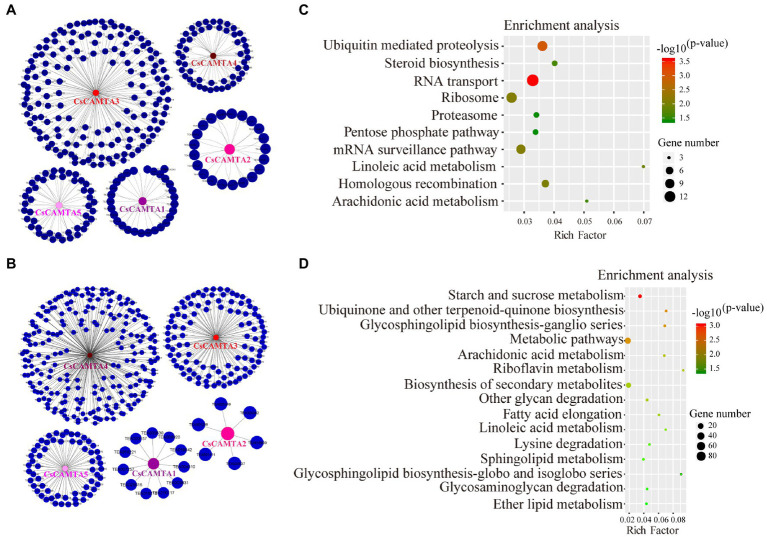
*CsCAMTA* gene co-expression network and the KEGG enrichment analysis of *CsCAMTA*s and their co-expressed genes under cold and PEG-simulated drought stress, respectively. **(A)** *CsCAMTA* gene co-expression network under cold stress; **(B)** *CsCAMTA* gene co-expression network under PEG-simulated drought stress; **(C)** KEGG enrichment analysis of *CsCAMTA*s and their positive co-expressed genes under cold stress; **(D)** KEGG enrichment analysis of *CsCAMTA*s and their positive co-expressed genes under PEG-simulated drought stress.

To further understand the function of *CsCAMTA* genes under cold and drought stresses, gene co-expression networks of *CsCAMTAs* were analyzed (Figures 6A,B; Supplementary Table S5). Our results showed that *CsCAMTA* genes had 300 and 401 positive co-expressed genes under cold and drought stress treatments, respectively. Under cold stress, the *CsCAMTA* gene co-expression network was constituted by 305 nodes and 300 edges. *CsCAMTA3* had the most number (156) of co-expressed genes, while *CsCAMTA2* had the least number (21) of co-expressed genes (Figure 6A; Supplementary Table S5). Under PEG-simulated drought treatment, there were 406 nodes and 401 edges in the *CsCAMTA* gene co-expression network. *CsCAMTA4* had the most number (223) of co-expressed genes, while *CsCAMTA2* had the least number (6) of co-expressed genes. In total, five co-expression networks were formed between *CsCAMTAs* and their co-expressed genes under both cold and drought stress treatments. No co-expression was seen between the five identified *CsCAMTA* genes (Figure 6B; Supplementary Table S5).

*CsCAMTAs* and their positive co-expressed genes were subjected to KEGG analysis to identify the enrichment of functional categories. Results showed that *CsCAMTAs* and their positive co-expressed genes were enriched in 10 metabolic pathways under cold treatment, including RNA transport, ubiquitin-mediated proteolysis, mRNA surveillance pathway, linoleic acid metabolism, steroid biosynthesis, arachidonic acid metabolism, etc. (Figure 6C; Supplementary Table S6). Under PEG-simulated drought stress, 15 metabolic pathways including starch and sucrose metabolism, ubiquinone, and other terpenoid-quinone biosynthesis, glycosphingolipid biosynthesis, arachidonic acid metabolism, biosynthesis of secondary metabolites, linoleic acid metabolism, lysine degradation, etc., were enriched by *CsCAMTAs* and the positive co-expressed genes (Figure 6D; Supplementary Table S6).

### Interaction Network of CsCAMTA Proteins in Tea Plant

**Figure 7:**
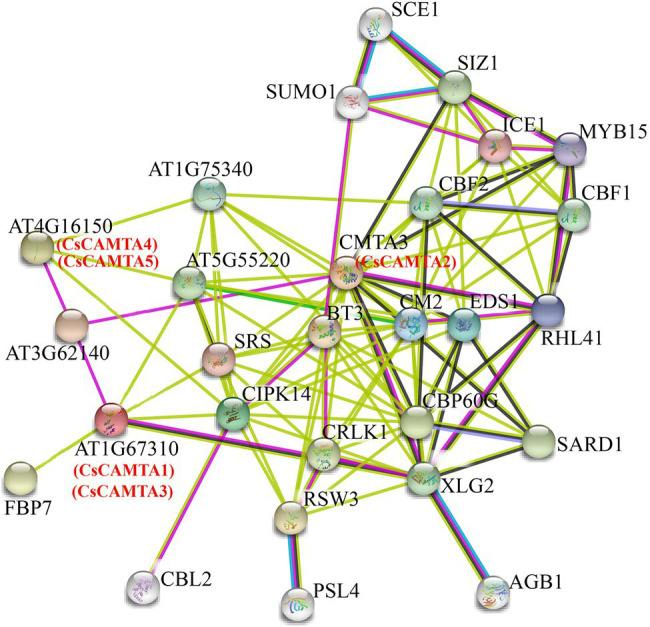
Putative interaction networks of CsCAMTA proteins in tea plant according to their orthologs in *Arabidopsis*. The Arabidopsis proteins and the homologous proteins in tea plant are displayed in black and red characters, respectively.

To understand the functional mechanisms of CsCAMTA proteins, protein interaction network analysis was done by using STRING software. Results showed that the five CsCAMTA proteins corresponded to three *Arabidopsis* homologs and had 25 potential interacting proteins (Figure 7). CsCAMTA2 corresponded to CMTA3, CsCAMTA4, and CsCAMTA5 corresponded to AT4G16150, CsCAMTA1, and CsCAMTA3 corresponded to AT1G67310 (Figure 7; Supplementary Table S7). Further analysis demonstrated that CMTA3 (CsCAMTA2) and AT4G16150 (CsCAMTA4 and CsCAMTA5), respectively, had the most (19) and the least (4) number of interacting proteins, while AT1G67310 (CsCAMTA1 and CsCAMTA3) had six interacting proteins. The CMTA3 (CsCAMTA2) interacting proteins including 11 transcription factors (such as CBF, CBP60G, RHL41, MYB15, ICE1, etc.), two calcium-related protein kinases (CIPK14, CRLK1), and 6 functional proteins (such as EDS1, CM2, XLG2, etc.). As to AT1G67310 (CsCAMTA1 and CsCAMTA3) and AT4G16150 (CsCAMTA4 and CsCAMTA5), two of their interacting proteins were transcription factors. AT4G16150 (CsCAMTA4, CsCAMTA5) and AT1G67310 (CsCAMTA1, CsCAMTA3) could also interact with CIPK14. It was also seen that AT3G62140, coding a NEFA-interacting nuclear protein, could interact with AT4G16150 (CsCAMTA4, CsCAMTA5), AT1G67310 (CsCAMTA1, CsCAMTA3), and CAMT3 (CsCAMTA2; Figure 7; Supplementary Table S7).

Studies have demonstrated that CAMTA transcription factor bound with the (G/A/C)CGCG(C/G/T) or (A/C)CGTGT *cis* element in the target gene promoter to regulate their expression (Noman et al., 2019; Sun et al., 2020). To further investigate the interaction network of CsCAMTA proteins in tea plant, promoter sequences of the 25 potential interacting protein homolog-coding genes in tea plant and those of the *CsCAMTA* genes were analyzed. Results revealed that the (G/A/C)CGCG(C/G/T) *cis* element was existed in the promoters of nine tea plant genes which coding proteins were homologous to AT1G67310.1, CBL2, SUMO1, CBF2, SARD1, MYB15, RHL41, AT4G16150.1, and XLG2 in the protein interaction network (Figure 7; Supplementary Table S8). The (A/C)CGTGT *cis* element was identified in the promoters of seven tea plant genes homologous to those coding CRLK1, CBF1, AT1G75340, CMTA3, SARD1, SCE1, and AT4G16150.1 (Supplementary Table S8). (A/C)CGTGT and (G/A/C)CGCG(C/G/T) were also, respectively, found in the promoter of *CsCAMTA2* and *CsCAMTA3*, and both were identified in the promoter of *CsCAMTA5* (Supplementary Table S8).

## Discussion

Tea plant is a kind of leafy economic crop and is vulnerable to external stresses (Zhang et al., 2017; Wu et al., 2018; Li et al., 2019; Zhou et al., 2019a). To do investigation on genes those function in leaf development regulation and stress resistance will be very important for quantity increment and quality improvement of tea. CAMTAs are important players in plant stress response as well as in plant development (Zhang et al., 2014; Yang et al., 2015; Furio et al., 2020). Although studies on CAMTA have been reported in many plant species, identification of CAMTA in tea plant lags behind. Here, genome-wide identification of *CAMTA* gene was first done, and five *CsCAMTA* genes were identified from the tea plant genome (Table 1). The number of *CAMTA* genes in tea plant was similar to that in *Arabidopsis* (6), *Oryza sativa* (6), *Zea mays* (7), *Medicago truncatula* (7), *citrus clementina* (4), and *Vitis vinifera* (4), but smaller than that in *Glycine max* (14) and *Brassica rapa* (9). It was consistent with the 4–8 *CAMTA* gene number identified in higher flowering plant species, although the tea plant genome size was, respectively, more than nine-fold and three-fold larger than that of *Brassica rapa* and *Glycine max* (Rahman et al., 2016; Wei et al., 2018; Xia et al., 2019). The result may reflect the evolutionary conservation of *CAMTA* gene in higher plants and a high ratio of repetitive sequences in tea plant genome.

CsCAMTA proteins displayed conserved characters in tea plant. The amino acid size of CsCAMTA proteins was ranged from 864 to 1,419, and all of the CsCAMTAs were predicted as nuclear localization proteins, which were similar to those reported in other plants (Table 1; Yang et al., 2015; Yue et al., 2015; Pant et al., 2018; Büyük et al., 2019; Meer et al., 2019). The typical CAMTA protein contains CG-1, TIG/IPT, ANK, IQ, and CaMBD domains. It was also reported that non-TIG/IPT domain CAMTA protein appeared in newly evolved flowering land plants (Rahman et al., 2016; Pant et al., 2018; Büyük et al., 2019). In our study, all the identified CsCAMTA proteins own the TIG/IPT domain (Figure 1B), indicating CsCAMTA proteins emerged before the divergence of flowering plants and non-flowering plants. IQ and CaMBD domains can interact with calmodulin in Ca^2+^-independent and Ca^2+^-dependent pathway, respectively; typical CAMTA protein always has two IQ domains, and the quantity of IQ domain is reported to affect CAMTA protein clustering in phylogenetic tree (Rahman et al., 2016; Pant et al., 2018). The five identified CsCAMTA proteins have both IQ and CaMBD domains (Figure 1B), suggesting that they can interact with calmodulin either there is Ca^2+^ or not. Phylogenetic analysis showed that CsCAMTA proteins were clustered in three different groups, which may be related to the IQ domain number and/or the relative position of IQ domain to CaMBD domain (Figure 1B). Gene structure analysis showed that most of the *CsCAMTA* genes had 10–13 introns, which was consistent with 12 introns found in most of the vascular plants (Figure 1A; Rahman et al., 2016; Kakar et al., 2018; Saeidi et al., 2019). *CsCAMTA2* had 19 introns, which was more than the most intron number (15) identified in other vascular plants (Figure 1A; Rahman et al., 2016; Pant et al., 2018). Intron, existing in eukaryotic genome, is believed important for gene expression regulation and for new functional protein generation (Chorev and Carmel, 2012; Wu et al., 2019). The high intron number may be related to the multifunction of *CsCAMTA2* in tea plant, although function study of *CsCAMTA2* is lacking. *AtCAMTA3*, the homologous gene of *CsCAMTA2* in *Arabidopsis*, was displayed important roles in freezing tolerance, SA mediated immune response, insect feeding response, hypersensitive response, etc. (Doherty et al., 2009; Laluk et al., 2012; Yue et al., 2015).

CAMTAs are important regulators of plant growth and development. AtCAMTA1 and 5 can regulate pollen development; tobacco NtER1 and Arabidopsis AtCAMTA3 were involved in ethylene-induced senescence process (Yang et al., 2015; Zhang et al., 2019a). Homologous phylogenetic analysis showed that CsCAMTA2, AtCAMTA1, and AtCAMTA3 were in group I, CsCAMTA4, CsCAMTA5, and AtCAMTA5 were in group III (Figure 2), which suggested that CsCAMTA2 might function in both senescence process and pollen development, and CsCAMTA4, 5 might function in regulating pollen development. Considering high expression of *CsCAMTA2* and lower expression of *CsCAMTA4* and *CsCAMTA5* in flower (Figures 4A, 5A), *CsCAMTA2*, *CsCAMTA4,* and *CsCAMTA5* may involve in pollen development by different regulating mechanisms. Expression analysis also showed that most of the *CsCAMTAs* had higher expression level in leaf tissues, and *CsCAMTA1* and *5* displayed relative lower expression in young and mature leaves and higher expression in old leaves (Figures 4A, 5A). Whether *CsCAMTA1* and *5* have function in regulating leaf senescence is worth investigating. Because tea plant is a kind of leaf-type economic crop, keeping leaf tender and delaying senescence of leaf is important for tea flavor and economic value improvement (Wu et al., 2018).

Studies also demonstrated that CAMTAs were crucial for stress response (Kim et al., 2013, 2020; Kakar et al., 2018). Our results showed that *CsCAMTA2* gene was responsive to cold, salt, and PEG-simulated drought treatments, as well as MeJA treatment (Figures 4, 5). Phylogenetic analysis showed that CsCAMTA2 was in group I, together with AtCAMTA1, AtCAMTA2, and AtCAMTA3 (Figure 2). In *Arabidopsis*, research showed that AtCAMTA1 played an important role in drought resistance; AtCAMTA3 was involved in cold response, pathogen defense response, resistance to insect herbivory, etc. (Yang et al., 2015; Pant et al., 2018). AtCAMTA2 was reported to function in metal stress detoxification and osmotic stress responses (Shen et al., 2015). These results suggested that the *CsCAMTA2* gene might be important for both biotic and abiotic stress responses of tea plant. According to the phylogenetic analysis, CsCAMTA1 and 3 were clustered together with AtCAMTA4 in group II (Figure 2). Gene expression analysis showed that the transcriptional levels of *CsCAMTA1* and *3* were obviously changed after cold, salt, or PEG-simulated drought treatments, suggesting that *CsCAMTA1* and *3* were involved in the response to these stresses. Although function of *AtCAMTA4* was not clear at present, research showed that expression of the *AtCAMTA4* homologs such as *CitCAMTA3* in *citrus* (*Ciclev10030636m*; Zhang et al., 2019a), *PtCAMTA1* and *7* in *Populus* (Wei et al., 2017), and *Pvul-CAMTA3* and *8* in *Phaseolus vulgaris* (Büyük et al., 2019), was significantly affected by salt and dehydration, cold, and salt stress, respectively. Recent study also showed that the *AtCAMTA4* homolog *TaCAMTA4* negatively regulated the basic resistance to leaf rust fungus in wheat (Wang et al., 2019). OsCAMTA1 (LOC_Os01g69910), clustered in group II with AtCAMTA4, CsCAMTA1 and 3, was reported important for cold resistance (Kim et al., 2013; Yue et al., 2015). CsCAMTA4 and 5 were clustered with AtCAMTA5 and 6 in group III (Figure 2). Our study showed that expression of *CsCAMTA4* and *5* was response to cold, salt, PEG-simulated drought, and MeJA treatments (Figures 4, 5), although *AtCAMTA5* and *6* were reported to function in plant development regulation (Shkolnik et al., 2019; Zhang et al., 2019a). *Citrus* CitCAMTA4 (Ciclev10004273m) and poplar PtCAMTA4 and 6 were clustered with AtCAMTA5 and 6 by phylogenetic analysis (Figure 2). The expression of *CitCAMTA4* (Ciclev10004273m) and *PtCAMTA4* and *6* was, respectively, regulated by salt and dehydration, cold, and salt stress (Wei et al., 2017; Büyük et al., 2019; Zhang et al., 2019a), suggesting that *CsCAMTA4* and *5* may also involve in stress response. Further analysis demonstrated that various stress-responsive *cis* elements were laid out in the promoters of *CsCAMTA* genes (Figure 3; Supplementary Table S3), supporting the role of *CsCAMTAs* in stress response.

Function annotation supports conserved function of CsCAMTAs in tea plant. By homologous annotation using TrEMBL, Swissprot, and Nr databases, all of the five *CsCAMTA* genes were annotated as calmodulin-binding transcription activators (Supplementary Table S4). GO and KEGG analysis showed that *CsCAMTA1*, *3* and *4* were annotated as lipase, ester hydrolase, LPA acyltransferase, and signal recognition and involved in transcription process (Supplementary Table S4). It has reported that CAMTA is important Ca^2+^ signal transducer, and can regulate the lipase-coding gene *EDS1* and *PAD4* expression, which are important for cold resistance and SA-mediated immune response (Yue et al., 2015; Kim et al., 2020). LPA acyltransferase was ABA responsive and induced by salt and drought stresses, and it was important for female gametophyte development in *Arabidopsis* (Kim et al., 2005; Aboagla and Hong, 2017). CsCAMTA5 was involved in transcription regulation and annotated as abnormal spindle-like microcephaly-associated protein, or myosin by GO and KEGG (Supplementary Table S4), suggesting that CsCAMTA5 was related to development and molecular mobility. Research demonstrated that calmodulin could regulate mobility of myosin V (Nguyen and Higuchi, 2005). CsCAMTA2 was also annotated as MLO-like protein and involved in stress response by GO and KEGG (Supplementary Table S4). These results indicated that CsCAMTA played important roles in signal transduction, development, and biotic and abiotic stress response in tea plant, which was the same as the function of CAMTA identified in other plants. There was no co-expression between the identified *CsCAMTA* genes (Figures 6A,B; Supplementary Table S5), indicating the *CsCAMTA* genes had different spatiotemporal expression models, or different biological functions. Cold and drought are two kinds of environmental stresses that tea plant is vulnerable to encounter during the life span (Liu et al., 2016; Li et al., 2019). The KEGG analysis suggested that *CsCAMTA* genes involved in cold response mainly by hormone regulation (such as linoleic acid metabolism, steroid biosynthesis, and arachidonic acid metabolism), transcriptional regulation (such as RNA transport and mRNA surveillance pathway), and protein processing (such as ubiquitin mediated proteolysis, ribosome, and proteasome; Figure 6C; Supplementary Table S6). Enrichment of these pathways was also shown in *Haloxylon ammodendron*, *Anthurium*, *Ricinus communis,* and other plants under cold stress (Tian et al., 2013; Eremina et al., 2016; Peng et al., 2019). Under PEG-simulated drought stress, pathways like hormone metabolism (including linoleic acid metabolism, and arachidonic acid metabolism), lipid metabolism (including sphingolipid metabolism, glycosphingolipid biosynthesis, fatty acid metabolism, ether lipid metabolism, etc.), carbon metabolism (including starch and sucrose metabolism, and glycan degradation) were enriched by *CsCAMTAs* and their co-expressed genes (Figure 6D; Supplementary Table S6). These pathways were also enriched in drought stressed *Cynanchum thesioides*, *Eruca vesicaria*, *Vicia sativa*, and *Glycine max* (Huang et al., 2019; Zhang et al., 2019b; Min et al., 2020). Under cold or drought stress, research showed that *CAMTA-*regulated genes always enriched in the similar pathways as described above (Kim et al., 2013; Pandey et al., 2013). These results suggested CsCAMTAs were involved in stress response by conserved regulating mechanism, and the transcriptome data was valuable for *CsCAMTA* gene function analysis in tea plant.

Protein interaction analysis showed that 57.9% of the CsCAMTA2-interacting proteins, 50% of the CsCAMTA4 and CsCAMTA5-interacting proteins, and 33.3% of the CsCAMTA1 and CsCAMTA3-interacting proteins were DNA binding proteins (Figure 7; Supplementary Table S7), suggesting that transcriptional regulation might be the main way of CsCAMTA mediated functional regulation. It is clear that ICE1, CBF1, and CBF2 are key regulators in cold and drought stresses (Kim et al., 2013; Zhou et al., 2019b). SARD1, EDS1, and CBP60G are involved in SA synthesis and thus important for SA mediated immunity response (Kim et al., 2013). RSW3, XLG2, and CIPK14 are important regulators in plant development and stress response (Heo et al., 2012; Ren et al., 2017; Lia et al., 2019). So, by interacting with those proteins, CsCAMTA played various roles in biotic and abiotic stresses response, as well as in growth and development. CAMTA can bind to the (G/A/C)CGCG(C/G/T) or the (A/C)CGTGT *cis* element in promoter region of the target genes (Yue et al., 2015; Sun et al., 2020). Our analysis showed that there were 16 tea plant homologous genes to those of *Arabidopsis* in the interaction network had the (G/A/C)CGCG(C/G/T) or the (A/C)CGTGT *cis* element in their promoter regions. In the promoter regions of *TEA023367.1* (*SARD1* gene homolog in tea plant) and *CsCAMTA5*, both (G/A/C)CGCG(C/G/T) and (A/C)CGTGT *cis* elements were identified (Supplementary Table S8). These results indicated that the interaction network in *Arabidopsis* may also exist in tea plant, and CsCAMTAs can interact with those *Arabidopsis* homologs in tea plant. It also showed that the (G/A/C)CGCG(C/G/T) or the (A/C)CGTGT *cis* element was laid in the promoter of *CsCAMTA2* and *3*, suggesting that *CsCAMTAs* may regulate a biological event by cooperation. In *Arabidopsis*, it demonstrated that *CAMTA1*, *2* and *3* were function together to suppress SA biosynthesis and to enhance freezing tolerance (Kim et al., 2013; Yang et al., 2015). In the interaction network, AT3G62140 can interact with AT4G16150 (CsCAMTA4, CsCAMTA5), AT1G67310 (CsCAMTA1, CsCAMTA3), and CAMT3 (CsCAMTA2; Figure 7; Supplementary Table S7); this result suggested that the *CsCAMTAs* can regulate some biological processes together. Further studies by using transgenic plant materials or biochemical assays will provide more evidences for *CsCAMTA* gene function identification and the interaction network of CsCAMTA proteins.

## Data Availability Statement

Publicly available datasets were analyzed in this study. This data can be found here: The cold acclimation RNA-Seq data are available in the NCBI’s.Sequence Read Archive (SRA) database (accession number: SRP108833). For NaCl and PEG treatments are available from the European Nucleotide Archive database (ENA; https://www.ebi.ac.uk/ena/browser/home) under project number accession PRJEB11522. For MeJA available in the NCBI SRA (sequence read archive, http://www.ncbi.nlm.nih.gov/sra/) respository under the accession number of SRP060335. For different tissues that was accessed from pcsb.ahau.edu.cn:8080/CSS/ (data downloading with user name 20170705F16HTSCCKF2479, password rwn160912abc).

## Author Contributions

QZ and HY contributed to conceptualization, supervision, and funding acquisition. MZ and FX performed project administration. FX, GM, and YW performed formal analysis. GM and YW done data curation. YW and YD contributed to methodology. YD and MN performed visualization and provided software. MZ and HY was involved in investigation. FX and MN performed validation. QZ and GM performed writing—original draft. FX and HY performed writing—review and editing. All authors contributed to the article and approved the submitted version.

## Funding

The work was supported by the National Key Research and Development Program of China (2021YFD1601103), the National Natural Science Foundation of China (no. U1404319), Scientific and Technological Research Projects of Henan Province (202102110230, 212102110399, and 222102110238), Key Scientific Research Projects of Universities in Henan Province (22A210023), the Training Program for Young Backbone Teachers in Colleges and Universities of Henan Province (2021GGJS100), Nanhu Scholars Program for Young Scholars of XYNU (2016060), and the National Training Programs of Innovation and Entrepreneurship for Undergraduates (202110477011).

## Conflict of Interest

The authors declare that the research was conducted in the absence of any commercial or financial relationships that could be construed as a potential conflict of interest.

## Publisher’s Note

All claims expressed in this article are solely those of the authors and do not necessarily represent those of their affiliated organizations, or those of the publisher, the editors and the reviewers. Any product that may be evaluated in this article, or claim that may be made by its manufacturer, is not guaranteed or endorsed by the publisher.

## Footnotes

^1^ http://pcsb.ahau.edu.cn:8080/CSS/

^2^ http://www.ncbi.nlm.nih.gov/cdd

^3^ http://smart.embl-heidelberg.de/

^4^ http://web.expasy.org/compute_pi/

^5^ http://web.expasy.org/protparam/

^6^ http://cello.life.nctu.edu.tw/

^7^ http://planttfdb.gao-lab.org/

^8^ http://myhits.isb-sib.ch/cgi-bin/motif_scan

^9^ http://bioinformatics.psb.ugent.be/webtools/plantcare/html/

^10^ https://string-db.org/cgi/input.pl?sessionId=3MKiLYehXbcT&input_page_show_search=on

## References

[ref1] Aboagla A. A. A. Hong Y. (2017). Lysophosphatidic acid Acyltransferase2 (LPAT2) enhances abscisic acid response and plays a positive role in osmotic stress in rice. J. Cell Sci. Ther. 8:1 (Suppl). doi: 10.4172/2157-7013.C1.039

[ref2] Bouché N. Scharlat A. Snedden W. Bouchez D. Fromm H. (2002). A novel family of calmodulin-binding transcription activators in multicellular organisms. J. Biol. Chem. 277, 21851–21861. doi: 10.1074/jbc.M200268200, PMID: 11925432

[ref3] Büyük İ. İlhan E. Şener D. Özsoy A. U. Aras S. (2019). Genome-wide identification of CAMTA gene family members in *Phaseolus vulgaris* L. and their expression profiling during salt stress. Mol. Biol. Rep. 46, 2721–2732. doi: 10.1007/s11033-019-04716-8, PMID: 30843175

[ref4] Chen C. J. Chen H. Zhang Y. Thomas H. R. Frank M. H. He Y.. (2020). TBtools: an integrative toolkit developed for interactive analyses of big biological data. Mol. Plant 13, 1194–1202. doi: 10.1016/j.molp.2020.06.009, PMID: 32585190

[ref5] Choi M. S. Kim M. C. Yoo J. H. Moon B. C. Koo S. C. Park B. O.. (2005). Isolation of a calmodulin-binding transcription factor from rice (*Oryza sativa* L.). J. Biol. Chem. 280, 40820–40831. doi: 10.1074/jbc.M504616200, PMID: 16192280

[ref6] Chorev M. Carmel L. (2012). The function of introns. Front. Genet. 3:55. doi: 10.3389/fgene.2012.00055, PMID: 22518112 PMC3325483

[ref7] Doherty C. J. Van Buskirk H. A. Myers S. J. Thomashow M. F. (2009). Roles for *Arabidopsis* CAMTA transcription factors in cold regulated gene expression and freezing tolerance. Plant Cell 21, 972–984. doi: 10.1105/tpc.108.063958, PMID: 19270186 PMC2671710

[ref8] Eremina M. Rozhon W. Poppenberger B. (2016). Hormonal control of cold stress responses in plants. Cell. Mol. Life Sci. 73, 797–810. doi: 10.1007/s00018-015-2089-6, PMID: 26598281 PMC11108489

[ref9] Furio R. N. Martínez-Zamora G. M. Salazar S. M. Coll Y. Perato S. M. Martos G. G.. (2020). Role of calcium in the defense response induced by brassinosteroids in strawberry plants. Sci. Hortic. 261:109010. doi: 10.1016/j.scienta.2019.109010

[ref10] Heo J. B. Sung S. Assmann S. M. (2012). Ca^2+^-dependent GTPase, extra-large G protein 2 (XLG2), promotes activation of DNA-binding protein related to vernalization 1 (RTV1), leading to activation of floral integrator genes and early flowering in *Arabidopsis*. J. Biol. Chem. 287, 8242–8253. doi: 10.1074/jbc.m111.317412, PMID: 22232549 PMC3318724

[ref11] Huang B. L. Li X. Liu P. Ma L. Wu W. Zhang X.. (2019). Transcriptomic analysis of *Eruca vesicaria* subs. Sativa lines with contrasting tolerance to polyethylene glycol-simulated drought stress. BMC Plant Biol. 19:419. doi: 10.1186/s12870-019-1997-2, PMID: 31604421 PMC6787972

[ref12] Kakar K. U. Nawaz Z. Cui Z. Cao P. Jin J. Shu Q.. (2018). Evolutionary and expression analysis of *CAMTA* gene family in *Nicotiana tabacum* yielded insights into their origin, expansion and stress responses. Sci. Rep. 8, 10322–10314. doi: 10.1038/s41598-018-28148-9, PMID: 29985386 PMC6037683

[ref13] Kim Y. Gilmour S. J. Chao L. Park S. Thomashow M. F. (2020). *Arabidopsis* CAMTA transcription factors regulate pipecolic acid biosynthesis and priming of immunity genes. Mol. Plant 13, 157–168. doi: 10.1016/j.molp.2019.11.001, PMID: 31733370

[ref14] Kim H. U. Li Y. Huang A. H. (2005). Ubiquitous and endoplasmic reticulum–located lysophosphatidyl acyltransferase, LPAT2, is essential for female but not male gametophyte development in *Arabidopsis*. Plant Cell 17, 1073–1089. doi: 10.1105/tpc.104.030403, PMID: 15772283 PMC1087987

[ref15] Kim Y. Park S. Gilmour S. J. Thomashow M. F. (2013). Roles of CAMTA transcription factors and salicylic acid in configuring the low-temperature transcriptome and freezing tolerance of *Arabidopsis*. Plant J. 75, 364–376. doi: 10.1111/tpj.12205, PMID: 23581962

[ref16] Laluk K. Prasad K. V. Savchenko T. Celesnik H. Dehesh K. Levy M.. (2012). The calmodulin-binding transcription factor SIGNAL RESPONSIVE1 is a novel regulator of glucosinolate metabolism and herbivory tolerance in *Arabidopsis*. Plant Cell Physiol. 53, 2008–2015. doi: 10.1093/pcp/pcs143, PMID: 23072934 PMC3516851

[ref17] Li Y. Y. Wang X. W. Ban Q. Y. Zhu X. X. Jiang C. J. Wei C. L.. (2019). Comparative transcriptomic analysis reveals gene expression associated with cold adaptation in the tea plant *Camellia sinensis*. BMC Genomics 20:624. doi: 10.1186/s12864-019-5988-3, PMID: 31366321 PMC6670155

[ref18] Lia A. Gallo A. Marti L. Roversi P. Santino A. (2019). EFR-mediated innate immune response in *Arabidopsis thaliana* is a useful tool for identification of novel ERQC modulators. Genes 10:15. doi: 10.3390/genes10010015, PMID: 30591693 PMC6357087

[ref19] Liu S. C. Xu Y. X. Ma J. Q. Wang W. W. Chen W. Huang D. J.. (2016). Small RNA and degradome profiling reveals important roles for microRNAs and their targets in tea plant response to drought stress. Physiol. Plant. 158, 435–451. doi: 10.1111/ppl.12477, PMID: 27282332

[ref20] Meer L. Mumtaz S. Labbo A. M. Khan M. J. Sadiq I. (2019). Genome-wide identification and expression analysis of calmodulin-binding transcription activator genes in banana under drought stress. Sci. Hortic. 244, 10–14. doi: 10.1016/j.scienta.2018.09.022

[ref21] Min X. Lin X. Ndayambaza B. Wang Y. Liu W. (2020). Coordinated mechanisms of leaves and roots in response to drought stress underlying full-length transcriptome profiling in *Vicia sativa* L. BMC Plant Biol. 20, 165–121. doi: 10.1186/s12870-020-02358-8, PMID: 32293274 PMC7161134

[ref22] Nguyen H. Higuchi H. (2005). Motility of myosin V regulated by the dissociation of single calmodulin. Nat. Struct. Mol. Biol. 12, 127–132. doi: 10.1038/nsmb894, PMID: 15665867

[ref23] Noman M. Jameel A. Qiang W. D. Ahmad N. Liu W. C. Wang F. W.. (2019). Overexpression of *GmCAMTA12* enhanced drought tolerance in *Arabidopsis* and soybean. Int. J. Mol. Sci. 20:4849. doi: 10.3390/ijms20194849, PMID: 31569565 PMC6801534

[ref24] Novikova D. D. Cherenkov P. A. Sizentsova Y. G. Mironova V. V. (2020). metaRE R package for meta-analysis of transcriptome data to identify the *cis*-regulatory code behind the transcriptional reprogramming. Genes 11:634. doi: 10.3390/genes11060634, PMID: 32526881 PMC7348973

[ref25] Ouyang Z. G. Mi L. F. Duan H. H. Hu W. Chen J. M. Peng T.. (2019). Differential expressions of *citrus* CAMTAs during fruit development and responses to abiotic stresses. Biol. Plant. 63, 354–364. doi: 10.32615/bp.2019.041

[ref26] Pandey N. Ranjan A. Pant P. Tripathi R. K. Ateek F. Pandey H. P.. (2013). CAMTA1 regulates drought responses in *Arabidopsis thaliana*. BMC Genomics 14:216. doi: 10.1186/1471-2164-14-216, PMID: 23547968 PMC3621073

[ref27] Pant P. Iqbal Z. Pandey B. K. Sawant S. V. (2018). Genome-wide comparative and evolutionary analysis of calmodulin-binding transcription activator (CAMTA) family in *Gossypium* species. Sci. Rep. 8, 1–17. doi: 10.1038/s41598-018-23846-w, PMID: 29615731 PMC5882909

[ref28] Peng M. C. Wang Y. L. Wang M. Chu G. (2019). Transcriptome profiling of *Haloxylon ammodendron* seedling at low temperature condition. Appl. Ecol. Environ. Res. 17, 1411–1429. doi: 10.15666/aeer/1701_14111429

[ref29] Qiu Y. Xi J. Du L. Suttle J. C. Poovaiah B. W. (2012). Coupling calcium/calmodulin-mediated signaling and herbivore-induced plant response through calmodulin-binding transcription factor AtSR1/CAMTA3. Plant Mol. Biol. 79, 89–99. doi: 10.1007/s11103-012-9896-z, PMID: 22371088

[ref30] Rahman H. Yang J. Xu Y. P. Munyampundu J. P. Cai X. Z. (2016). Phylogeny of plant CAMTAs and role of AtCAMTAs in nonhost resistance to *Xanthomonas oryzae* pv. Oryzae. Front. Plant Sci. 7:177. doi: 10.3389/fpls.2016.00177, PMID: 26973658 PMC4770041

[ref31] Ren Y. Li Y. Jiang Y. Wu B. Miao Y. (2017). Phosphorylation of WHIRLY1 by CIPK14 shifts its localization and dual functions in *Arabidopsis*. Mol. Plant 10, 749–763. doi: 10.1016/j.molp.2017.03.011, PMID: 28412544

[ref32] Saeidi K. Zare N. Baghizadeh A. Asghari-Zakaria R. (2019). *Phaseolus vulgaris* genome possesses *CAMTA* genes, and *phavuCAMTA1* contributes to the drought tolerance. J. Genet. 98:31. doi: 10.1007/s12041-019-1069-2 30945676

[ref33] Shangguan L. Wang X. Leng X. Liu D. Ren G. Tao R.. (2014). Identification and bioinformatics analysis of signal responsive/calmodulin-binding transcription activators gene models in Vitis Vinifera. Mol. Biol. Rep. 41, 2937–2949. doi: 10.1007/s11033-014-3150-5, PMID: 24458826

[ref34] Shen C. Yang Y. Du L. Wang H. (2015). Calmodulin-binding transcription activators and perspectives for applications in biotechnology. Appl. Microbiol. Biotechnol. 99, 10379–10385. doi: 10.1007/s00253-015-6966-6, PMID: 26450508

[ref35] Shi J. Ma C. Qi D. Lv H. Yang T. Peng Q.. (2015). Transcriptional responses and flavor volatiles biosynthesis in methyl jasmonate-treated tea leaves. BMC Plant Biol. 15:233. doi: 10.1186/s12870-015-0609-z, PMID: 26420557 PMC4588909

[ref36] Shkolnik D. Finkler A. Pasmanik-Chor M. Fromm H. (2019). Calmodulin-binding transcription activator 6: a key regulator of Na^+^ homeostasis during germination. Plant Physiol. 180, 1101–1118. doi: 10.1104/pp.19.00119, PMID: 30894419 PMC6548231

[ref37] Sun T. Huang J. Xu Y. Verma V. Jing B. Sun Y.. (2020). Redundant CAMTA transcription factors negatively regulate the biosynthesis of salicylic acid and N-hydroxypipecolic acid by modulating the expression of *SARD1* and *CBP60g*. Mol. Plant 13, 144–156. doi: 10.1016/j.molp.2019.10.016, PMID: 31733371

[ref38] Tamura K. Stecher G. Peterson D. Filipski A. Kumar S. (2013). MEGA6: molecular evolutionary genetics analysis version 6.0. Mol. Biol. Evol. 30, 2725–2729. doi: 10.1093/molbev/mst197, PMID: 24132122 PMC3840312

[ref39] Tian D. Q. Pan X. Y. Yu Y. M. Wang W. Y. Zhang F. Ge Y. Y.. (2013). *De novo* characterization of the *Anthurium* transcriptome and analysis of its digital gene expression under cold stress. BMC Genomics 14:827. doi: 10.1186/1471-2164-14-827, PMID: 24267953 PMC4046746

[ref40] Trapnell C. Williams B. A. Pertea G. Mortazavi A. Kwan G. Van Baren M. J.. (2010). Transcript assembly and quantification by RNA-Seq reveals unannotated transcripts and isoform switching during cell differentiation. Nat. Biotechnol. 28, 511–515. doi: 10.1038/nbt.1621, PMID: 20436464 PMC3146043

[ref41] Wang Y. Wei F. Zhou H. Liu N. Niu X. Yan C.. (2019). TaCAMTA4, a Calmodulin-interacting protein, involved in defense response of wheat to *Puccinia triticina*. Sci. Rep. 9, 1–8. doi: 10.1038/s41598-018-36385-1, PMID: 30679453 PMC6345913

[ref42] Wang G. Zeng H. Hu X. Zhu Y. Chen Y. Shen C.. (2015). Identification and expression analyses of calmodulin-binding transcription activator genes in soybean. Plant and Soil 386, 205–221. doi: 10.1007/s11104-014-2267-6

[ref420] Wei C. Yang H. Wang S. Zhao J. Liu C. Gao L.. (2018). Draft genome sequence of *Camellia sinensis* var. sinensis provides insights into the evolution of the tea genome and tea quality. Proc. Natl. Acad. Sci. U. S. A. 115, E4151–E4158. doi: 10.1073/pnas.1719622115 29678829 PMC5939082

[ref43] Wei M. Xu X. Li C. (2017). Identification and expression of CAMTA genes in *Populus trichocarpa* under biotic and abiotic stress. Sci. Rep. 7:17910. doi: 10.1038/s41598-017-18219-8, PMID: 29263356 PMC5738416

[ref44] Wu J. Li A. Cai H. Zhang C. Lei C. Lan X.. (2019). Intron retention as an alternative splice variant of the cattle *ANGPTL6* gene. Gene 709, 17–24. doi: 10.1016/j.gene.2019.05.031, PMID: 31102716

[ref45] Wu Z. J. Ma H. Y. Zhuang J. (2018). iTRAQ-based proteomics monitors the withering dynamics in postharvest leaves of tea plant (*Camellia sinensis*). Mol. Genet. Genomics 293, 45–59. doi: 10.1007/s00438-017-1362-9, PMID: 28852881

[ref46] Xia E. H. Li F. D. Tong W. Li P. H. Wu Q. Zhao H. J.. (2019). Tea plant information archive: a comprehensive genomics and bioinformatics platform for tea plant. Plant Biotechnol. J. 17, 1938–1953. doi: 10.1111/pbi.13111, PMID: 30913342 PMC6737018

[ref47] Yang T. Peng H. Whitaker B. D. Conway W. S. (2012). Characterization of a calcium/calmodulin-regulated *SR/CAMTA* gene family during tomato fruit development and ripening. BMC Plant Biol. 12:19. doi: 10.1186/1471-2229-12-19, PMID: 22330838 PMC3292969

[ref48] Yang Y. Sun T. Xu L. Pi E. Wang S. Wang H.. (2015). Genome-wide identification of *CAMTA* gene family members in *Medicago truncatula* and their expression during root nodule symbiosis and hormone treatments. Front. Plant Sci. 6:459. doi: 10.3389/fpls.2015.00459, PMID: 26150823 PMC4472986

[ref49] Yue R. Lu C. Sun T. Peng T. Han X. Qi J.. (2015). Identification and expression profiling analysis of calmodulin-binding transcription activator genes in maize (Zea mays L.) under abiotic and biotic stresses. Front. Plant Sci. 6:576. doi: 10.3389/fpls.2015.00576, PMID: 26284092 PMC4516887

[ref50] Zhang Q. Cai M. C. Yu X. M. Wang L. S. Guo C. F. Ming R.. (2017). Transcriptome dynamics of *Camellia sinensis* in response to continuous salinity and drought stress. Tree Genet. Genomes 13:78. doi: 10.1007/s11295-017-1161-9

[ref51] Zhang L. Du L. Poovaiah B. W. (2014). Calcium signaling and biotic defense responses in plants. Plant Signal. Behav. 9:e973818. doi: 10.4161/15592324.2014.973818, PMID: 25482778 PMC4623097

[ref52] Zhang D. Han Z. Li J. Qin H. Zhou L. Wang Y.. (2020). Genome-wide analysis of the SBP-box gene family transcription factors and their responses to abiotic stresses in tea (*Camellia sinensis*). Genomics 112, 2194–2202. doi: 10.1016/j.plaphy.2013.05.021, PMID: 31870711

[ref53] Zhang J. Pan X. Ge T. Yi S. Lv Q. Zheng Y.. (2019a). Genome-wide identification of *citrus CAMTA* genes and their expression analysis under stress and hormone treatments. J. Hortic Sci. Biotech. 94, 331–340. doi: 10.1080/14620316.2018.1504631

[ref54] Zhang X. Yang Z. Li Z. Zhang F. Hao L. (2019b). *De novo* transcriptome assembly and co-expression network analysis of *Cynanchum thesioides*: identification of genes involved in resistance to drought stress. Gene 710, 375–386. doi: 10.1016/j.gene.2019.05.055, PMID: 31200084

[ref55] Zhou Q. Y. Cheng X. Wang S. S. Liu S. R. Wei C. L. (2019a). Effects of chemical insecticide imidacloprid on the release of C6 green leaf volatiles in tea plants (*Camellia sinensis*). Sci. Rep. 9, 1–6. doi: 10.1038/s41598-018-36556-0, PMID: 30679494 PMC6345918

[ref56] Zhou Q. Han Y. Pan J. Yuan H. Li X. Qin M.. (2019b). Research progress in plant cold resistance mechanism. J. Xinyang Normal Univ. (Natural Sci. Edn.) 3, 511–516 (in Chinese with English Abstract). doi: 10.3969/j.issn.1003-0972.2019.03.031

